# Giant trochanteric pressure sore: Use of a pedicled chimeric perforator flap for cover

**DOI:** 10.4103/0970-0358.53025

**Published:** 2009

**Authors:** Sandeep Mehrotra

**Affiliations:** Reconstructive Surgery Centre, Command Hospital (Eastern Command), Alipore, Kolkata - 700 027, India

**Keywords:** Pedicled perforator chimeric flap, Pressure sore, Trochanteric sore

## Abstract

Pressure sores are increasing in frequency commensurate with an ageing population with multi-system disorders and trauma. Numerous classic options are described for providing stable wound cover. With the burgeoning knowledge on perforator anatomy, recent approaches focus on the use of perforator-based flaps in bedsore surgery. A giant neglected trochanteric pressure sore in a paraplegic is presented. Since conventional options of reconstruction appeared remote, the massive ulcer was successfully managed by a chimeric perforator-based flap. The combined muscle and fasciocutaneous flaps were raised as separate paddles based on the anterolateral thigh perforator branches and provided stable cover without complications. Perforators allow versatility in managing complex wounds without compromising on established principles.

## INTRODUCTION

Pressure sore reconstruction is among the most challenging procedures, having arguably the highest complication rates. Patients require significant nursing effort, prolonged hospitalization, and expensive supportive therapy. A multimodality approach involving several pre, intra, and postoperative considerations minimize adverse outcomes.

Wounds acquired from pressure over bony prominences are described since antiquity, their etiology based on tissue ischemia with subsequent necrosis. In 1938, Davis was the first to suggest a flap to replace the unstable scar.[1] Presently, reconstruction includes radically removing necrotic bone, stump padding, and dead space obliteration with muscle using an adequately mobilized large flap to avoid tension while preserving subsequent options of reconstruction in case of a recurrence.

Conventional approaches employ muscle, fasciocutaneous, or composite myocutaneous flaps or skin flaps that are based on random vascular inputs. Recent approaches are based on locally available perforators and offer significant freedom of approach with simple operative procedures.[2] Trochanteric pressure sores are half as common as sacral or ischial sores and typically have minimal skin loss. Occasionally, neglected patients may present with severe tissue loss and mandate unconventional approaches.

## CASE REPORT

**Figure 1:**
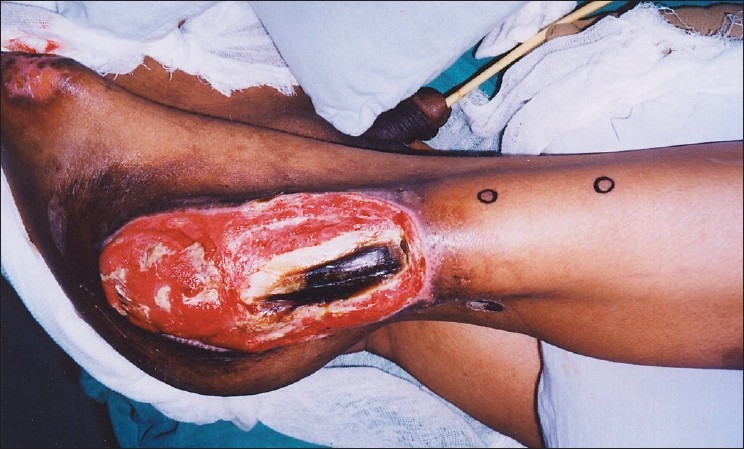
Giant right trochanteric pressure sore with exposed trochanters and gangrenous tensor fascia lata. Doppler identified perforators are marked

A 57-year-old electrician, a native of Ladakh, had fallen from an electric pole and sustained a fracture of his seventh dorsal vertebra resulting in paraplegia. He was referred to our center 2 months after management elsewhere. On admission, he was in a poor general condition with anemia (Hb 8 g%) and high fever with spikes up to 104°F. There was clinical and radiological evidence of hypostatic pneumonia. An examination revealed a giant right trochanteric sore measuring 24 × 14 cms [Figure 1]. The trochanter was protruding through the wound and the tensor fascia was frankly gangrenous. He had local wound sepsis with osteomyelitis and pus discharge from the exposed trochanter and clinical septicemia. X-rays confirmed osteomyelitis of the greater trochanter. Further investigations revealed low serum proteins with albumin of 2.5 g% and normal renal function tests. Broad spectrum antibiotic cover with Inj Cefotaxime and Amikacin was started and 2 units of blood were transfused. Nutritional support was given through Nasogastric tube feeds. These were prepared using raw eggs blended in milk along with commercially available enteral nutritional support to provide 3 gm/kg protein and 3000 Kcal/day. Nasogastric feeds were given at 3 ml/min round the clock and the patient was allowed an oral diet as per desire. Physiotherapists, nursing staff, and ancillary staff ensured general patient hygiene and chest and limb physiotherapy with care of the bowel, bladder, and skin. Over the ensuing week, there was improvement in the patient's general condition with a reduction of fever, clearing of respiratory congestion, and an increase in hemoglobin to 10.4g%. Despite a reduction of discharge from the pressure sore there was no discernable healing.

**Figure 2:**
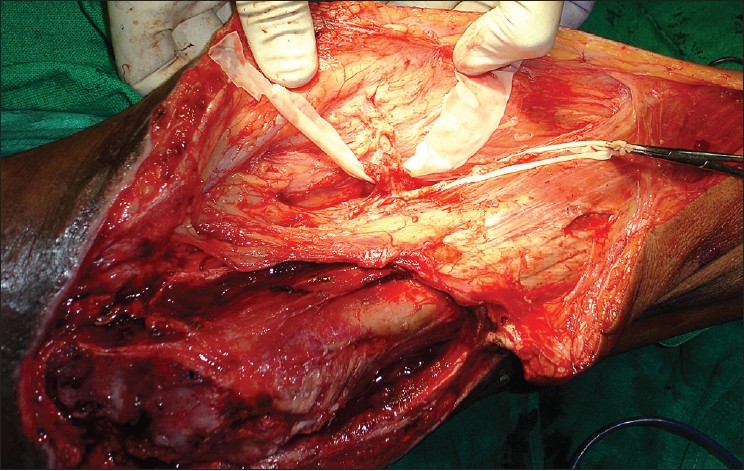
Lateral circumflex femoral artery with branches to skin and vastus lateralis dissected and taped

**Figure 3:**
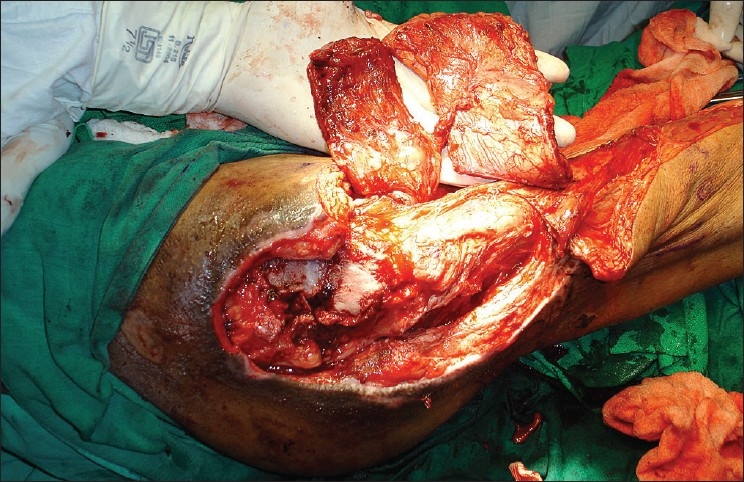
Paddles of vastus (proximal) and skin (distal) raised on perforator branches

**Figure 4:**
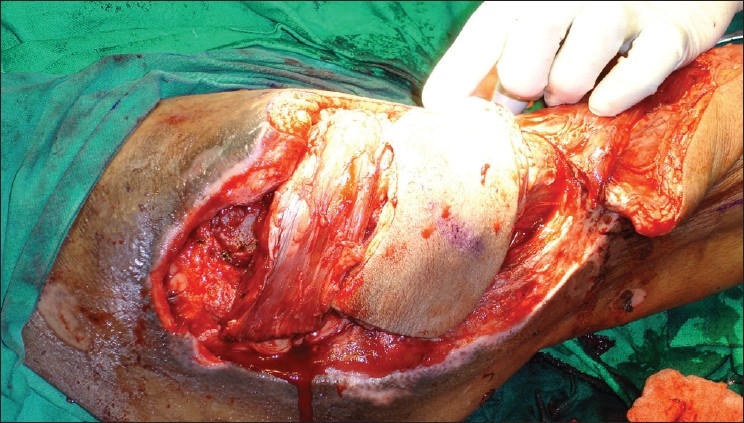
Muscle component placed proximally over the debrided trochanteric area and fasciocutaneous component placed distally over femur shaft

Early surgery was planned after stabilization of his general condition. Preoperative portable Doppler marking of the anterolateral thigh perforator was done and was suggestive of two terminal prominent branches [Figure 1]. Surgical exploration revealed necrosis of a significant portion of the tensor fascia lata (TFL) and the proximal third vastus lateralis. There was osteomyelitis of the greater and lesser trochanter with purulent collection. Radical debridement and shaving of unhealthy bone was done after safeguarding the perforator. A defect of 28 × 16 cms was created with 15 cms in length of the upper femur left exposed. Immediate single stage reconstruction with an anterolateral thigh flap was planned and the perforator was dissected. This revealed an intact branch to the middle third of the vastus lateralis apart from the skin supply [Figure 2]. A chimeric flap was pedicled on the lateral circumflex femoral artery using the anterolateral thigh fasciocutaneous and vastus lateralis muscle component on separate perforator branches [Figure 3]. Proximal dissection of the vessels realized a pedicle length of 14 cms, which permitted easy rotation and inset of the vastus over the upper femur and trochanteric raw area and the muscle was covered by split skin graft. The fasciocutaneous component was inset over the residual distal raw area. The donor site could be primarily closed except a 6 × 2 cms strip over the distal extent, which was also covered by skin graft [Figure 4]. Thorough wound irrigation with hydrogen peroxide, povidone iodine, and saline with meticulous hemostasis was ensured. No drains were employed and 2 units of fresh blood were administered perioperatively.

**Figure 5:**
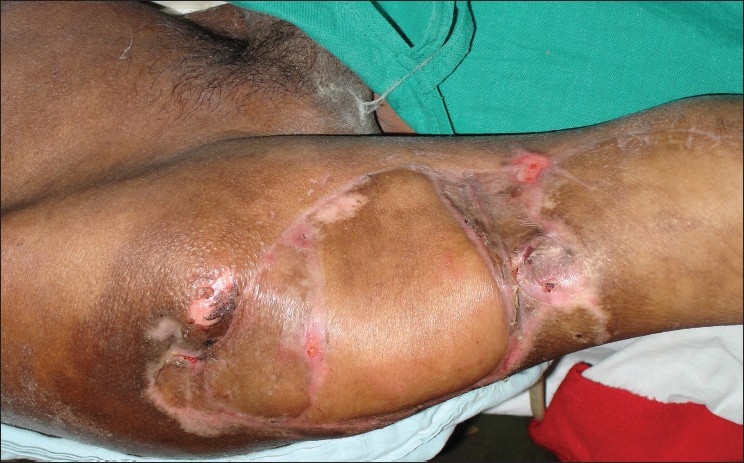
Well consolidated flaps 2 months after the surgery

After the surgery, the patient had a rapid recovery and was afebrile after the second postoperative day. He was nursed in the supine and right lateral position on an air mattress until healing and graft consolidation. Post-operative physiotherapy, nursing, and dietary care was continued. Antibiotics were stepped down to oral cephalosporins before being discontinued after 2 weeks. The flap survived completely with no local complications or residual raw areas. The patient was allowed to lie on the right hip after 6 weeks for short periods under supervision, which were gradually increased. At a 2-month follow-up visit, the flaps were stable with no recurrence of ulceration [Figure 5].

## DISCUSSION

Pressure sores are assuming increasing importance due to an ageing population and higher incidence of neurological trauma with 3–10% bed occupancy in large hospitals being attributed to this cause. Patients need dedicated nursing, entail longer hospitalization, and incur higher costs. The increasing demands on the system require improvements in surgical care for Grade 3 and 4 ulcers with early recovery and rehabilitation. The basic principles involve radical debridement, obliteration of dead space, and padding of exposed bone by muscle while preserving alternative options for any recurrences. Individual full-time patient attendants, dedicated and trained nurses, physiotherapists, dieticians, and ancillary staff are available at our center and are important for holistic care. Trochanteric pressure sores have been traditionally managed by the TFL myocutaneous flap based on the lateral circumflex femoral artery as described by Nahai.[3] Modifications include the retro position V-Y flap and the bipedicled TFL flap. Recent research into perforator anatomy has opened new vistas into pressure sore reconstruction. Multiple flap options with various perforator-based approaches are being employed for common pressure ulcer sites.[4]

The proximal pedicled anterolateral thigh flap and its myocutaneous variant employing the vastus lateralis have been described by Tzeng for trochanteric defects with reliable results.[5] Lee, *et al.* have described the extended use of these flaps for ischial sores.[6] These flaps were employed as fasciocutaneous or myocutaneous variants by the authors. The present case had a nearly one foot long extensive defect with significant bone and soft tissue necrosis, which was not amenable to conventional approaches. Lemaire, *et al.* have advised the limited use of free flaps in cases where defects cannot be covered by pedicled approaches.[7] Long operative times in sick patients, requirement of microsurgical equipment, and expertise often preclude this option. Though described as a microvascular transfer by Cavadas,[8] this chimeric flap has been employed successfully as a pedicled transfer in this case.

The use of both anterolateral thigh skin and vastus lateralis muscle as separate paddles based on the lateral circumflex femoral artery branches was considered a viable option to limit both donor site and patient morbidity. Proximal dissection of the vascular pedicle afforded enough length to inset the muscle over the exposed trochanter and obliterate dead space. The skin paddle was employed to cover the distal extent of the defect. To prevent tension on the flaps, a strip of raw area over the distal extent was grafted along with the muscle. Being on the non weight bearing aspect, it posed minimal concern. The flaps healed well without wound complications. The patient recovered rapidly from sepsis with improvement in his general condition and had stable cover at discharge after 2 months. The presence of life-threatening sepsis in pressure sores can increase the risk of mortality to 55%.[9] Timely debridement and stable cover can salvage difficult cases. The use of perforator-based flaps permit a freedom of choice hitherto unavailable to reconstructive surgeons. Optimal results can be obtained without needless function or tissue sacrifice or compromising on established standards.
